# Tunable structured laser over full spatial spectrum

**DOI:** 10.1038/s41377-026-02243-3

**Published:** 2026-03-13

**Authors:** Quan Sheng, Jing-Ni Geng, Jia-Qi Jiang, Tian-Chang Liu, David James Spence, Helen Margaret Pask, Allam Srinivasa Rao, Takashige Omatsu, Shi-Jie Fu, Zhen-Xu Bai, Zhi-Wei Lv, Wei Shi, Jian-Quan Yao, Carmelo Rosales-Guzmán, Zhi-Han Zhu

**Affiliations:** 1 State Key Laboratory of Precision Measuring Technology and Instruments, School of Precision Instrument of Opto-Electronics Engineering, Tianjin University, Tianjin, 300072 China; 2 Institute of Quantum Science and Technology, and Department of Physics, Yanbian University, Yanji, 133002 Jilin China; 3 Wang Da-Heng Center, Heilongjiang Key Laboratory of Quantum Control, Harbin University of Science and Technology, Harbin, 150080 China; 4 MQ Photonics Research Centre, School of Mathematical and Physical Sciences, Macquarie University, Sydney, NSW 2109 Australia; 5 Molecular Chirality Research Center, Chiba University, 1-33 Yayoi-cho, Inage-ku, Chiba 263-8522 Japan; 6 Center for Advanced Laser Technology, Hebei University of Technology, Tianjin, 300401 China; 7 Centro de Investigaciones en Óptica, A.C., Loma del Bosque 115, Colonia Lomas del Campestre, 37150 León, GTO Mexico

**Keywords:** Solid-state lasers, Photonic devices

## Abstract

Modern tunable lasers are indispensable instruments in fundamental science and optical frequency applications. With the growing interest in structured light, there’s a need for tunable structured lasers with high transverse electromagnetic (TEM) modal purity across a broad spatial spectrum, but no commercial solutions exist. Here, we present the first tunable structured laser that can emit all possible single TEM modes with extremely high purity over a wide range. This is achieved by the collaborative use of intracavity pump geometry and astigmatic detuning to selectively gain a desired mode while blockading others. Our astigmatic oscillator can tunably generate any TEM mode within the spatial bandwidth, achieving over 40,000 orthogonal Hermite-Gauss modes in the experiment, and each can be further expanded into numerous Hermite-Laguerre-Gauss modes via unitary transformation. This approach does not require extra intracavity beam shaping components, offering a promising design for commercially available structured lasers with full spatial spectrum tunability.

## Introduction

Tunable lasers with wavelength controllable emission constitute a critical infrastructure in modern optical science and its applications^1–3^. Over the past 60 years, significant advancements have been made in commercial technologies for single-longitudinal-mode lasers that enable tuning across a broad frequency range without mode hopping, i.e., a continuous evolution of the optical frequency, such as widely used Ti-sapphire and distributed feedback semiconductor lasers^4,5^. The fundamental principle behind frequency tunability involves introducing appropriate intracavity *dispersion* and *geometry* to break the frequency symmetry of propagation and gain behaviors within a broad gain resonator, thereby discriminating the desired longitudinal mode from others^6^. Traditionally, resonators were designed to operate in their fundamental transverse electromagnetic (TEM) mode to facilitate easier longitudinal-mode selection and enhance pump efficiency. However, this paradigm has been shifting over the past decade due to the rise of studying structured light and optical orbital angular momentum (OAM)^7–16^. The primary focus of the new emerging field is the generation and application of various structured light. Consequently, recent laser community shows great enthusiasm for structured lasers capable of producing specific or tunable higher-order transverse modes directly from a cavity^17–23^. However, there are currently no commercial technologies or practical solutions available for achieving output of all possible single TEM modes over a wide tunable range of spatial spectrum.

Compared to frequency tuning within the one-dimensional continuous variable space, tunability in the spatial spectrum necessitates two-dimensional (2D) modal control and selection within the discrete variable space defined by Hermite-Gauss (HG) or Laguerre-Gauss (LG) mode families^24^. Both TEM modal families can resonate within optical cavities through phase conjugation loops, exhibiting pattern invariance during each trip^25^. Therefore, the prevailing strategy for achieving tunability in the spatial spectrum primarily focuses on introducing modal-sensitive gain via pump geometry, ensuring that the desired mode reaches the lasing threshold while suppressing other modes. Perhaps due to broader interest in OAM, significant efforts have been directed towards structured lasers within the LG family^8,17–23^. However, the LG approach typically requires sophisticated pump shaping and struggles to achieve single-TEM mode output due to the lack of effective helicity symmetry breaking^17^. In contrast, the more accessible off-axis pump technique can be employed to build up intracavity spatial wavefunctions with ‘HG-like’ patterns^26,27^. Because of two underlying mechanisms:

(1) it provides a narrow spatial gain linewidth for the desired mode, resulting in a lasing threshold that exhibits HG modal discrimination depending on the lateral displacement; and (2) during each passing through the laser medium, the local amplitude gain induced by the pump slightly affects the purity of amplified HG mode, i.e., a kind of self-healing characteristic, allowing for a high effective net gain; see *Principles* for details.

Nevertheless, due to unwanted gain competition between one-dimensional (1D) and 2D modes that share the same optimal pump position, the tunable range of this commercially viable approach is restricted to the 1D HG space, and the modal purity declining rapidly for higher-order outputs^28^.

In this study, we present the first widely tunable structured laser that offers comprehensive access to the 2D spatial spectrum without mode hopping. By introducing suitable astigmatic detuning to break the rotational symmetry of intracavity diffraction—analogous to the role of dispersion in frequency tuning—we blockade modal gain for unwanted competitors, thereby overcoming the limitation on 2D spectrum access imposed by the off-axis pump approach and achieving extremely high modal purity. To validate this principle, we experimentally demonstrate tunable generation of arbitrary single TEM modes within the spatial bandwidth, specifically over 40,000 distinct HG modes, using such an astigmatic oscillator.

## Results

### Spatial spectrum in Hermite-Laguerre-Gauss modal space

**Fig. 1:**
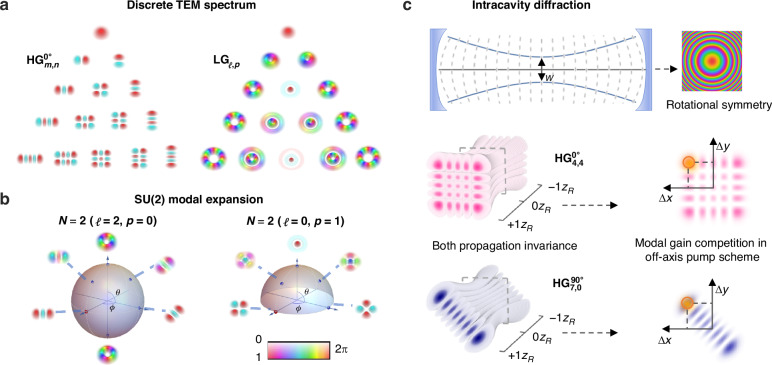
Concept of structured laser defined in Hermite-Laguerre-Gauss modal space. **a** All possible single HG and LG patterns with orders no more than 4 at the waist plane. **b** Generalized HLG patterns on SU(2) modal spheres for *N* *=* 2. **c** 3D field profiles of two representative modes, ${{\rm{HG}}}_{\mathrm{7,0}}^{90^{\circ}}$ and ${{\rm{HG}}}_{\mathrm{4,4}}^{0^{\circ}}$, during resonance within a rotation-symmetry cavity, which have the same peak position and thus forming gain competition

Two best-known TEM families, Hermite- and Laguerre-Gauss modes, are complete eigen sets of the paraxial wave equation (PWE) in Cartesian and cylindrical coordinates, respectively, and their spatial wavefunctions at the waist plane (*z*=0) are given by^8^:

1 $${{\rm{HG}}}_{m,n}^{{\boldsymbol{\phi }}=0}(\mathop{r}\limits^{ \rightharpoonup})=\sqrt{\frac{{2}^{1-n-m}}{\pi n!m!}}\frac{1}{w}{{{\rm{e}}}^{-\frac{{x}^{2}+{y}^{2}}{{w}^{2}}}H}_{m}\left(\frac{\sqrt{2}x}{w}\right){H}_{n}\left(\frac{\sqrt{2}y}{w}\right)$$

and

2 $${\mathrm{LG}}_{{\mathcal{l}},p}(\mathop{r}\limits^{ \rightharpoonup })=\sqrt{\frac{2p!}{\pi \left(p+{\mathcal{l}}\right)!}}\frac{1}{w}{{{\rm{e}}}^{-\frac{{r}^{2}}{{w}^{2}}}\left(\frac{\sqrt{2}r}{w}\right)}^{\,\left|{\mathcal{l}}\right|}{{\rm{e}}}^{-i{\mathcal{l}}\varphi }{L}_{p}^{\left|{\mathcal{l}}\right|}\left(\frac{2{r}^{2}}{{w}^{2}}\right)$$

where ${H}_{m/n}\left(\cdot \right)$ and ${L}_{\left|{\mathcal{l}}\right|,p}\left(\cdot \right)$ are Hermite and Laguerre polynomials with bivariate modal indices $(m,n)$ and $({\mathcal{l}},p)$, and $w$ is the fundamental beam waist. Note that $\phi \in \left[\mathrm{0,2}\pi \right]$ represents the rotational angle of Cartesian coordinates defined by longitude of a modal sphere and, in particular, $\phi =0$ denotes $(x,y)$ coincides with horizontal and vertical directions. For a given modal order defined as $N=2p+\left|{\mathcal{l}}\right|=m+n$, there are total $N+1$ orthonormal HG and LG modes. Figure 1a shows all possible HG and LG modes with $N\le 4$. Despite sharing the same fundamental mode TEM_00_, two families have their own special features in transverse patterns determined by corresponding modal indices. Specifically, indices ${\mathcal{l}}$ (an integer) and $p$ (a positive integer) for LG modes determine numbers of azimuthal twisting (regarding OAM) and radial reversal in phase structures, respectively; while, for HG modes, positive integers $m$ and $n$ decide separately phase-reversal number along the $(x,y)$ coordinates *predefined* by $\phi$. Except for the ground order ($N=0$), modes in each order can form $\left(N+1\right)/2$ distinct Poincaré-like modal spheres^29,30^. For instance, Fig. 1b illustrates one and half such spheres for $N=2$. On these higher-order SU(2) modal spheres, states on equator are rotating ${{\rm{HG}}}_{m,n}^{\phi }$, poles are occupied by ${{\rm{LG}}}_{\pm {\mathcal{l}},p}$ conjugates following the relation ${\mathcal{l}}=m-n$, and other areas represent intermediate Hermite-Laguerre-Gauss (HLG) states defined by the group structure. All possible states on the same sphere, uniquely determined by the longitude ($\phi$) and latitude ($\theta$) angles, can be connected reciprocally via astigmatic unitary transformation^31,32^.

Namely, (1) one can access the full spatial spectrum of TEM modes using astigmatic modal conversion (AMC) of either HG or LG complete set; and (2) the tunable range and fineness of a structured laser can thus be characterized by available modal orders $N$ and indices $(m,n)$ or $({\mathcal{l}},p)$, respectively.

The complete three-dimensional spatial wavefunctions ${{\rm{HG}}}_{m,n}^{\phi }(\mathop{r}\limits^{ \rightharpoonup },z)$ and ${{\rm{LG}}}_{\pm {\mathcal{l}},p}(\mathop{r}\limits^{ \rightharpoonup },z)$ can be derived from the diffraction integral by employing Eqs. (1) and (2) as pupil functions. These beam profiles, see examples in Fig. 1c, exhibit propagation invariance, except for global scaling and the acquisition of *N*-dependent Gouy phases. This self-similar diffraction suggests that HG and LG maintain pattern invariance during resonance within optical cavities, forming the basis for higher-order mode generation through laser gain control capable of modal discrimination. Notably, a cylindrically symmetric cavity can resonate with a waist-matched set of LG modes; however, due to rotational symmetry, *it also accommodates HG sets across all possible rotating Cartesian coordinates*. The absence of restrictions on $\phi$, i.e., rotational symmetry breaking, prevents the use of the off-axis pump technique to generate 2D (bivariate) HG modes with both non-zero *m* and *n*, as will be demonstrated subsequently.

### Spatial modal gain by off-axis pump

The selection rule of HG cavity modes is governed by the local gain of the off-axis pump, facilitating the desired mode to achieve the lasing threshold prior to others. For a given HG cavity mode initially from the spontaneous radiation, the gross gain acquired from unit pump power is proportional to its spatial overlap with the pump, denoted as $P(\mathop{r}\limits^{ \rightharpoonup })$, within the gain medium, given by:

3 $$G(m,n)=\kappa \int {{\rm{HG}}}_{m,n}^{\phi }(\mathop{r}\limits^{ \rightharpoonup })P(\mathop{r}\limits^{ \rightharpoonup}){dV}$$

where $\kappa$ is the local gain factor describing the amplitude growth rate at the pump overlapping area, and the corresponding threshold pump power should be ${P}_{\mathrm{th}}(m,n)\propto 1/G(m,n)$^6^. According to the polynomial ${H}_{m/n}\left(\cdot \right)$ in Eq. (1), the maximum intensities of 1D and 2D HG modes occur at their respective outermost peaks, with 1D peaking at two ends and 2D modes at four corners. That is, an HG cavity mode with one of its peaks aligned with the Gaussian pump center achieves higher effective gain and a lower lasing threshold. However, this mechanism indicates that an inevitable gain competition exists between two specific yet representative modes in Fig. 1c—$\,{{\rm{HG}}}_{\mathrm{7,0}}^{90^\circ }$ and ${{\rm{HG}}}_{\mathrm{4,4}}^{0^\circ }$—because both exhibiting the same peak position at $\triangle x=\triangle y=1.75w$. Beyond displacement-sensitive gross modal gain, the ‘self-healing’ characteristic of HG modes also plays a pivotal role making the off-axis pump technique valid. This characteristic can be attributed to the higher net modal gain obtained during each oscillation, given by:

4 $$N\left(m,n\right)=\left\langle {{\rm{HG}}}_{m,n}^{\phi }(\mathop{r}\limits^{ \rightharpoonup})|{{\rm{g}}}_{m,n}^{\phi }(\mathop{r}\limits^{ \rightharpoonup})\right\rangle G(m,n)$$

where ${{\rm{g}}}_{m,n}^{\phi }\left(\mathop{r}\limits^{ \rightharpoonup }\right)$ denotes spatial complex amplitude of the mode experienced the local amplitude gain by off-axis pump; see Supplementary Information for details.

**Fig. 2:**
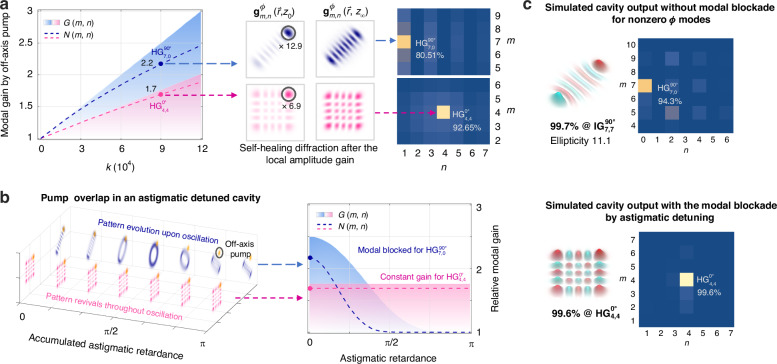
Principles of modal gain control by off-axis pump and modal blockade by astigmatic detuning. **a** Simulated single-pass gross (*G*) and net (*N*) gain for two competing modes (lefthand), ${{\rm{HG}}}_{\mathrm{7,0}}^{90^{\circ}}$ and ${{\rm{HG}}}_{\mathrm{4,4}}^{0^{\circ}}$, within a cylindrically symmetric cavity, with the pump spot size configured to $0.7w$, their pattern evolution from the origin (${z}_{0}$) to the Fourier (${z}_{\infty }$) planes after the local amplitude gain (middle), as well as associated density matrices (righthand). **b** Theoretical demonstration of modal blockade through intracavity astigmatism, where the continuous pattern variation of the undesired ${{\rm{HG}}}_{\mathrm{7,0}}^{90^{\circ}}$ results in its failure to compete in net gain with the ${{\rm{HG}}}_{\mathrm{4,4}}^{0^{\circ}}$. **c** Theoretical steady-state wavefunctions of cavity outputs, both in the absence and presence of astigmatic detuning for nonzero-$\phi$ modal blockade, where the spatial wavefunctions were simulated using Fox-Li iteration, and the corresponding density matrices were obtained through numerical projection on the ${{\rm{HG}}}_{m,n}^{0^{\circ}}$ space

The numerical analysis presented in Fig. 2a examines the modal gain and competition mechanism of the two competing HG modes. This analysis assumes that the outermost peak of each mode experiences the same local gain factor $\kappa$, leading to around double net modal gain during each trip. Specifically, the simulation shows that ${{\rm{HG}}}_{\mathrm{7,0}}^{90^\circ }$ and ${{\rm{HG}}}_{\mathrm{4,4}}^{0^\circ }$ obtain 2.2 (12.9) and 1.7 (6.9) times net modal (local amplitude) gains as setting *κ* = 9 × 10^4^, respectively. The findings indicate that the local gain offered by the off-axis pump does not substantially degrade modal purity, remaining 80.51% and 92.65% purity for the two modes, see spatial spectra displayed by density matrices in Fig. 2a right-hand, and thereby exhibiting diffraction self-healing, as shown in Fig. 2a middle. For that, the off-axis pump consistently provides effective net gain throughout cyclic oscillations. However, the inevitable gain competition between these two example modes also demonstrates that relying solely on off-axis pump gain control is impossible to generate 2D HG modes. More generally, when the maximum intensity patterns of 1D and 2D modes coincide, the latter is disadvantaged in gain competition due to lesser spatial overlap with the pump, thus restricting the tunable range always to the 1D HG modal space.

### Nonzero ${\boldsymbol{\phi }}$ mode blockade by astigmatic detuning

#### How to circumvent the limitation, or more specifically, to blockade modal gain for undesired competitors?

A natural consideration is seeking to reduce somehow the average pump spatial overlap for HG modes with nonzero $\phi$ throughout the oscillation process. Although this task appears challenging, the astigmatic unitary transformation mentioned previously offers a straightforward and viable solution, with which we do not even have to consider any complex active intracavity control. As demonstrated in Fig. 2b, the introduction of a subtle astigmatic detuning of ${\rm{\pi }}/q$ along the Cartesian coordinates with *ϕ* = 0 within a cavity that supports *q* cycles induces distinct diffraction behaviors in the competing mode pair ${{\rm{HG}}}_{\mathrm{7,0}}^{90^\circ }$ and ${{\rm{HG}}}_{\mathrm{4,4}}^{0^\circ }$. Due to the axially separable Gouy phase characteristic of HG modes, their beam profiles remain axially self-similar within the same Cartesian coordinates^30,31^. That is, the target 2D mode ${{\rm{HG}}}_{\mathrm{4,4}}^{0^\circ }$ consistently exhibits ‘pattern revival’ with each passage through the gain medium^33^, analogous to the transmission of a linear polarization state along a birefringence axis, thereby maintaining stable net modal gain. In contrast, owing to the intracavity astigmatic detuning, the pattern of the undesired ${{\rm{HG}}}_{\mathrm{7,0}}^{90^\circ }$ gradually transforms into that of the orthogonal one ${{\rm{HG}}}_{\mathrm{7,0}}^{-90^\circ }$ via the ${{\rm{LG}}}_{\mathrm{7,0}}$ mode during oscillations. This results in a significant loss in pump overlap and associated net gain, facilitating a modal blockade for nonzero *ϕ* HG spectra.

Consequently, the HG modal set in the astigmatic cavity is constrained to rectangular symmetry as defined by Cartesian coordinates with $\phi =0$, unlocking full spectrum tunability in HG space. In addition to the primary 1D competitor, it is noteworthy that the intracavity astigmatic detuning also blockade all possible undesired modes, potentially enhancing the output modal purity (or spatial linewidth). The theoretical steady-state wavefunctions of cavity output depicted in Fig. 2c, simulated using the Fox-Li iteration method with a pattern correlation threshold 99% for adjacent cycles, corroborate this inference. Specifically, the modal purity of the ${{\rm{HG}}}_{\mathrm{7,0}}^{90^\circ }$ from a cylindrically symmetric cavity without the modal blockade for nonzero-*ϕ* modes is approximately 94.3%, which is actually closer to the Ince-Gaussian mode ${{\rm{IG}}}_{\mathrm{7,7}}^{90^\circ }$ with an ellipticity of 11 (99.7% purity), whereas the desired ${{\rm{HG}}}_{\mathrm{4,4}}^{0^\circ }$ from the astigmatic detuned cavity exhibits a near-perfect purity of 99.6%. Here, the modal purity, for both the theory and following experiments, was characterized by the inner product of cavity outputs and their nearest eigen modes.

### Experimental setup

**Fig. 3:**
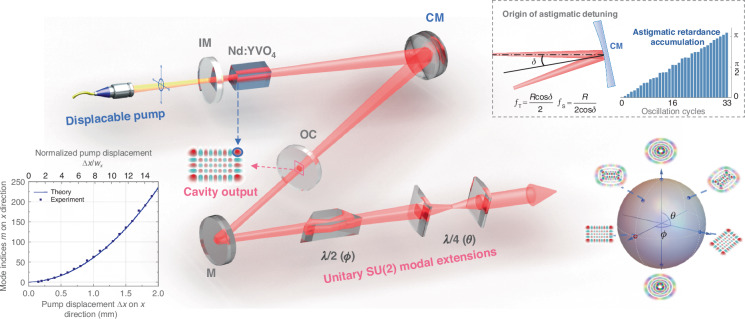
Schematic of the experimental setup, where are the input mirror (IM), Nd:YVO_4_ crystal, concave mirror (CM), output coupler (OC), mirror (M), and astigmatic retarders used for modal conversion composed by a Dove prism ($\lambda /2$) and cylindrical lens pair ($\lambda /4$). The left-bottom inset illustrates the theoretical lateral displacement of the pump center as a function for selecting modal indices $m$ and $n$, which is normalized to the fundamental waist of cavity modes. The upper-right inset illustrates the origin of astigmatic detuning and the corresponding retardance accumulation upon oscillations; further details are provided in “Methods”

Figure 3 depicts the experimental configuration designed to achieve a tunable single-transverse-mode laser throughout the HLG space. This setup incorporates an astigmatic oscillator operating at 1064 nm, which is capable of generating arbitrary ${{\rm{HG}}}_{m,n}^{0^\circ }$ modes, and an extra-cavity AMC for further unitary transformations of the modes as required.

The astigmatic oscillator is built using a V-shaped cavity arrangement with a folding angle of $\delta =\mathrm{10}^\circ$. The crucial astigmatic retardance originates from the use of a common curved mirror, CM, rather than an off-axis parabolic mirror avoiding astigmatism, to fold the cavity, resulting in different focal powers on the tangential (${f}_{T}=(R\cos \delta )/2$) and sagittal planes (${f}_{S}=R/(2\cos \delta )$). This precise oscillator design (see “Methods”), achieved without the need for additional astigmatic elements, can accumulate a total $\pi$ astigmatic retardance throughout the oscillation process, as shown in the upper-right inset. This intrinsic astigmatic detuning breaks the rotational (or cylindrical) symmetry of intracavity diffraction, thereby permitting the excitation of arbitrary 2D modes ${{\rm{HG}}}_{m,n}^{0^\circ }$ (here $\phi =0^\circ$ aligns with the table plane) through lateral displacement of the pump beam, as previously discussed.

The extra-cavity AMC comprises a Dove prism and a pair of cylindrical lenses, each having an identical focal length of $f=\mathrm{100}{\rm{mm}}$, serving as $\lambda /2(\phi )$ and $\lambda /4(\theta )$ astigmatic retarders, respectively. Additionally, a relay lens with a focal length of $f=\mathrm{100}{\rm{mm}}$ is utilized for waist matching (not depicted). The sequential arrangement of the $\lambda /2$ and $\lambda /4$ retarders facilitate the unitary rotation of the generated mode ${{\rm{HG}}}_{m,n}^{0^\circ }$, allowing access to all possible SU(2) coherent states on the modal sphere, i.e., generalized HLG modes^29–31^.

### Experimental results

The tunability of the oscillator within the spatial degrees of freedom defined by HG space, characterized by the accessible range of bivariate modal indices $m$ and $n$, was initially validated. In experiment, the pump center was initially aligned with the cavity axis, allowing the oscillator to operate in the fundamental mode ${{\rm{HG}}}_{\mathrm{0,0}}^{0^\circ }$. Using this position as a reference, higher-order modes ${{\rm{HG}}}_{m,n}^{0^\circ }$ were achieved by laterally displacing the pump center. As demonstrated in [Video-1], the modal indices $m$ and $n$ increase continuously with lateral displacements in the horizontal ($\varDelta x$) and vertical ($\varDelta y$) dimensions, respectively, while displacements in both dimensions yield a 2D modal output as anticipated.

In both dimensions, the maximum displacements of the pump center were constrained to approximately 1.9 mm by the crystal aperture, corresponding to a normalized displacement exceeding 14 times the fundamental cavity waist of 136 μm. According to the relationship depicted in the left-bottom inset of Fig. 3, the accessible modal order for both $m$ and *n* are exceeding 200. That is to say, by simply adjusting the pump position, it is possible to generate over 40,000 distinct HG modes from the oscillator on demand. Experimentally, the highest mode order achieved was $N=4\mathrm{30}$ when $\Delta x=\Delta y=1.9{\rm{mm}}$ under a 2 W pump power, although this mode ${{\rm{HG}}}_{\mathrm{214,216}}^{0^\circ }$ exhibited unequal $m$ and $n$. This observation accords with the fact that the cavity beam waist has a slightly elliptical profile.

**Fig. 4:**
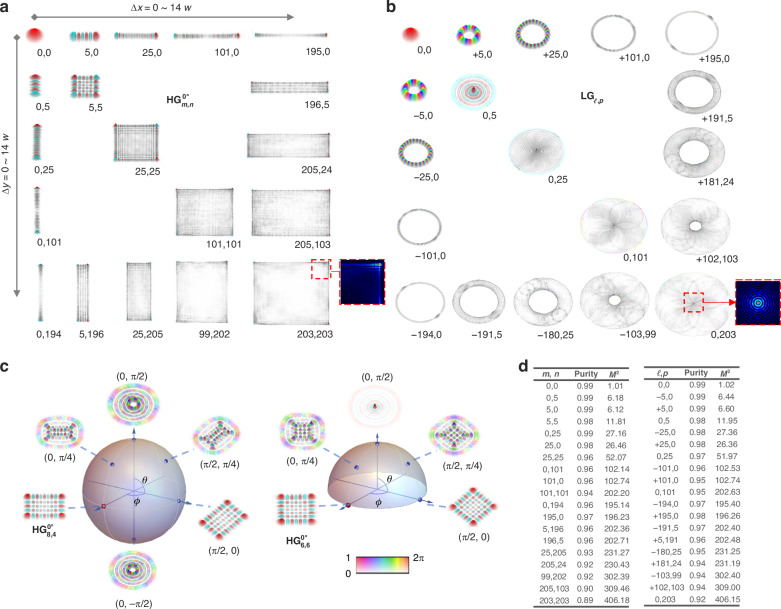
Experimental results for spatial tunability. Typical spatial complex amplitudes of **a** HG and **b** LG modes recorded in the experiment; corresponding intensity patterns are provided in additional data in Supplementary Information. **c** Generalized HLG modal extensions with ${{\rm{HG}}}_{\mathrm{8,4}}^{0^{\circ}}$ and ${{\rm{HG}}}_{\mathrm{6,6}}^{0^{\circ}}$ as inputs. **d** Modal purities and beam quality factors obtained from corresponding spatial complex amplitudes

To assess the quality of the single-transverse-mode output across the tunable range, we sampled several modes from ${{\rm{HG}}}_{\mathrm{0,0}}^{0^\circ }$ to ${{\rm{HG}}}_{\mathrm{20}\mathrm{3,20}3}^{0^\circ }$ using spatial complex amplitude measurements^34^. Figure 4a presents the observations, which display nearly perfect complex amplitude patterns. Beyond subjective qualitative visual assessment, these patterns provide comprehensive information about the laser fields and can be used for further quantitative modal characterization. Specifically, the modal purity indicated in the table in Fig. 4d was obtained via numerical projection measurement, while the beam quality ${M}^{2}$ values next to purities were calculated using numerical diffraction methods, further details on the characterization can be founded in Supplementary Information. Both characterizations confirmed the high modal quality of the oscillator output.

Subsequently, by employing the Dove prism to rotate the oscillator modes to $\phi =90^\circ$, the final output was transformed into ${{\rm{LG}}}_{{\mathcal{l}},p}$ after passing through the $\lambda /4$ astigmatic retarder, see [Video-2] demonstrating the continuous tunability in LG space. Figure 4b illustrates the measured spatial complex amplitudes, corresponding modal purity and beam quality ${M}^{2}$ are provided in Fig. 4d. In comparison to their HG counterparts, each LG mode exhibits a consistent Gouy phase and a corresponding ${M}^{2}$ value within the radial dimension. Furthermore, the conversion from 1D HG modes results in pure azimuthal modes with ${\mathcal{l}}=m$ or $n$; whereas, a 2D mode input with equal $m$ and $n$ generates pure radial modes that carry no net OAM. Beyond these point-to-point pairs of HG and LG outputs, by rotating both the $\lambda /2$ and $\lambda /4$ astigmatic retarders, each original oscillator mode can be further transformed into numerous ‘elliptical’ HLG modes, which can full cover the surface of their higher-order SU(2) modal sphere. Figure 4c presents two examples of such SU(2) modal extension with ${{\rm{HG}}}_{\mathrm{8,4}}^{0^\circ }$ and ${{\rm{HG}}}_{\mathrm{6,6}}^{0^\circ }$ as inputs, respectively. It is shown that, on the basis of a high-quality HG oscillator, new extending HLG modes via the SU(2) rotation also exhibit high beam quality.

**Fig. 5:**
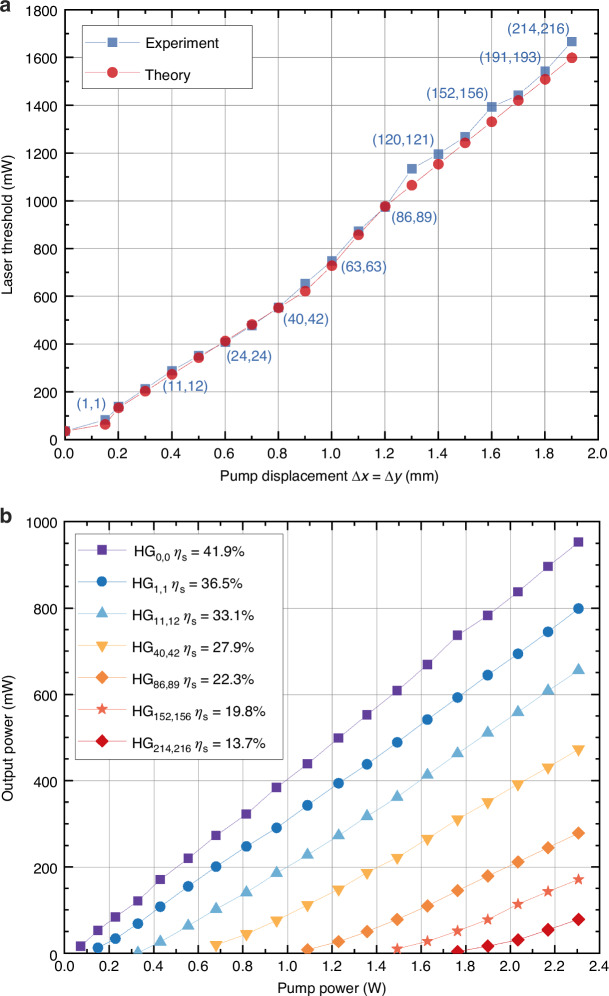
Lasing power behavior upon the operating spatial mode. **a** Laser threshold of typical 2D ${{\rm{HG}}}_{m,n}$ modes excited by off-axis pumping, the numbers in the bracket are the mode indices obtained in the experiment. **b** Laser output power of typical 2D ${{\rm{HG}}}_{m,n}$ modes as a function of input pump power

In a manner similar to the power-frequency dependence observed in conventional tunable lasers, the tunable structured laser presented in this study also exhibits a power dependence on the operating spatial mode. This phenomenon primarily arises from the fact that the pump overlap in Eq. (3) and the associated effective gain achieved in each oscillation diminish with increasing modal order. Figure 5a illustrates the measured mode-threshold dependence, which aligns well with theoretical predictions. Experimentally, the threshold was observed to initiate at 36 mW for the fundamental mode and progressively increased with pump displacement to generate higher-order modes. For instance, the thresholds for ${{\rm{HG}}}_{\mathrm{102,104}}^{0^\circ }$ and ${{\rm{HG}}}_{2\mathrm{14,2}\mathrm{16}}^{0^\circ }$ outputs reached 1.13 W and 1.67 W, respectively.

Figure 5b depicts the measured output power curve as a function of the incident pump power. The slope efficiency of the output modes gradually decreased from 41.9% to 22.3% and 13.7% as the modal order increased from ${{\rm{HG}}}_{\mathrm{0,0}}$ to ${{\rm{HG}}}_{8\mathrm{6,89}}$ and ${{\rm{HG}}}_{2\mathrm{14,216}}$, respectively. The output power of the highest order ${{\rm{HG}}}_{2\mathrm{14,216}}$ achieved 78 mW under an incident pump power of 2.3 W. Notably, both theoretical and experimental results mentioned above were obtained with a constant pump radius of 100 μm. Therefore, employing a variable pump radius to optimize the pump overlap and effective gain of higher-order modes can, in principle, mitigate the power and modal purity decline associated with increasing modal order. Besides, employing multi-edge pumps may be another feasible path to over such declines.

## Discussion

We have demonstrated the first tunable structured laser with the capability to fully access the spatial spectrum defined within the HLG space. This oscillator can tunably output over 40,000 distinct HG modes within the spatial bandwidth determined by system aperture, each of which can be further expanded into numerous HLG modes on its SU(2) modal sphere via unitary transformation. The underlying principle enabling the full spatial spectrum tunability involves the collaborative use of both off-axis pumping and astigmatic detuning. The off-axis pump provides a basic mode-dependent gain via pump geometry, while the astigmatic detuning introduces crucial modal blockade for all unwanted competing modes with nonzero *ϕ* by breaking the rotational symmetry of intracavity diffraction. That way, the spatial wavefunction of any desired HG mode can be built up exactly during the intracavity gain cycles, thereby paving the way towards the production of high-purity single TEM outputs over a wide tuning range.

In the experimental implementation, we engineered an ingenious V-shaped cavity possessing an intrinsic astigmatic detuning that accumulates a total $\pi$ retardance for the oscillating modes. This design ensures that HG modes, in coordinates defined by the astigmatism, consistently exhibit ‘pattern revival’ with each trip, thereby sustaining stable gain and Q-factor. Conversely, modes in undesired coordinates with nonzero $\phi$ undergo continuous pattern variation and are thus eliminated in the gain competition. As a result, this approach allows the off-axis pumping technique—the most accessible intracavity gain control—to access the full spatial spectrum. More important, this principle does not require additional active components for light structuring, thereby simplifying integration with existing laser technology frameworks and providing a genuine turnkey solution for the development of commercially available tunable structured lasers.

Beyond radiations from laser gain media, this intracavity beamforming mechanism can also be applied to nonlinear optics systems, such as optical parametric oscillators, Raman/Brillouin, and even air lasers^35–39^, achieving tunable generation of high-purity structured coherent and twin-photon beams. Furthermore, the theory illustrated in Fig. 2c suggests that, in the absence of the crucial nonzero $\phi$ modal blockade, the cavity tends to output 1D Ince-Gaussian modes. That is to say, the so-called ‘polygonal optical vortices’ identified in a recent study^40^ and originally reported in ref.^41^ are, in fact, the astigmatic transformations of Ince-Gaussian modes. This finding offers a new insight for studying Ince-Gaussian beams.

As the tunability over full spatial spectrum is unlocked, a variety of novel applications are anticipated, bring also specific performance requirements for the tunable structured laser. The first aspect that comes to mind is the rapid tuning speed, which facilitates swift encoding in communication and detection links, as well as the reconfiguration of light-matter interfaces. The tuning speed between eigenmodes is determined by the displacement speed of the pump; thus, digitally controlling the pump geometry could be a promising approach towards modal switching at hundreds and even kilohertz frequencies. Regarding tuning performance within the SU(2) subspace, although theoretically, a unitary transformation is lossless in both purity and energy, the experimental rotation of the Dove prism and cylindrical lens pair is cumbersome, particularly for higher-order modes. Exploiting flat optics with geometric phase can provide not only high conversion accuracy but also rapid modal switching via polarization dependence^16,31^. Another aspect worth exploring is the extension of the framework into the vector region, capable of generating arbitrary polarization patterns. This could facilitate potential applications in the emerging fields of topological structured light and vectorial light–matter interaction^42–46^.

## Materials and methods

### Experimental details

The cavity comprises a plano input mirror (IM), a plano-concave high-reflectivity mirror (CM) with a curvature radius ($R$) of 200 mm, and a plano output coupler (OC). Specifically, the ratio of intracavity equivalent focal lengths, with distances between IM and CM, and CM and OC being 130 mm and 170 mm, respectively, is ${f}_{T}:{f}_{S}=0.977$. This leads to a subtle astigmatic detuning of $0.03\pi$ per trip. The transmittance of OC is $T=3 \%$, enabling an average of approximately 33 oscillation cycles. The laser crystal used was an *a*-cut Nd:YVO_4_ crystal with a cross-section of 5 mm × 5 mm and a length of 8 mm. The crystal with a doping concentration of 0.5-at.% absorbed ~86% of the incident non-polarized pump. According to the ABCD matrix calculation, the fundamental mode ${{\rm{HG}}}_{\mathrm{0,0}}$ of the cavity should display a slightly elliptical profile of 136 μm (${w}_{x}$) by 137 μm (${w}_{y}$) within the crystal, necessitating an optimal pump radius of 100 μm. The non-polarized pump light at 878.6 nm, emitted by the laser diode, is delivered via a fiber with a core diameter of 105 μm and a numerical aperture (NA) of 0.22, resulting a 100 μm pump spot and a Rayleigh length of 0.8 mm in the crystal. The coupling optics for the pump light, attached to the fiber, are mounted on a translation stage, enabling precise control of the pump spot within the crystal with a resolution of 1 μm, while ensuring alignment parallel to the cavity axis.

## Supplementary information

Supplementary Document

Video 1

Video 2

## Data Availability

The data that support the findings of this study are available from the corresponding author upon reasonable request.

## References

[1] Duarte, F. J. *Tunable Laser Optics* 2nd edn (CRC Press, 2015).

[2] Freudiger, C. W. et al. Stimulated Raman scattering microscopy with a robust fibre laser source. *Nat. Photonics* **8**, 153–159 (2014). 25313312 10.1038/nphoton.2013.360 PMC4193905

[3] Zhang, Q. et al. Room-temperature near-infrared high-Q perovskite whispering-gallery planar nano lasers. *Nano Lett.* **14**, 5995–6001 (2014). 25118830 10.1021/nl503057g

[4] Yang, J. K. et al. Titanium: sapphire-on-insulator integrated lasers and amplifiers. *Nature* **630**, 853–859 (2024). 38926612 10.1038/s41586-024-07457-2

[5] Coldren, L. A. et al. Tunable semiconductor lasers: a tutorial. *J. Lightwave Technol.* **22**, 193–202 (2004).

[6] Siegman, A. E. *Lasers* Ch. 11, 432–440 (University Sciences Books, 1986).

[7] Forbes, A. et al. Structured light. *Nat. Photonics* **15**, 253–262 (2021).

[8] Allen, L. et al. Orbital angular-momentum of light and the transformation of Laguerre–Gaussian laser modes. *Phys. Rev. A* **45**, 8185–8189 (1992). 9906912 10.1103/physreva.45.8185

[9] Shen, Y. et al. Optical vortices 30 years on: OAM manipulation from topological charge to multiple singularities. *Light Sci. Appl.* **8**, 90 (2019). 31645934 10.1038/s41377-019-0194-2 PMC6804826

[10] Liu, X. et al. Spatiotemporal optical vortices with controllable radial and azimuthal quantum numbers. *Nat. Commun.* **15**, 5435 (2024). 38937504 10.1038/s41467-024-49819-4 PMC11211508

[11] Yao, A. M. & Padgett, M. J. Orbital angular momentum: origins, behavior and applications. *Adv. Opt. Photonics* **3**, 161–204 (2011).

[12] Wang, J. W. et al. Vectorial light-matter interaction–exploring spatially structured complex light fields. *AVS Quantum Sci.* **2**, 031702 (2020).

[13] Rubinsztein-Dunlop, H. et al. Roadmap on structured light. *J. Opt.* **19**, 013001 (2016).

[14] Goldberg, A. Z. et al. Quantum concepts in optical polarization. *Adv. Opt. Photonics* **13**, 1 (2020).

[15] Buono, W. T. & Forbes, A. Nonlinear optics with structured light. *Opto-Electron. Adv.* **5**, 210174 (2022).

[16] Dorrah, A. H. & Capasso, F. Tunable structured light with flat optics. *Science* **376**, eabi6860 (2022). 35446661 10.1126/science.abi6860

[17] Forbes, A. et al. Orbital angular momentum lasers. *Nat. Rev. Phys.* **6**, 352–364 (2024).

[18] Forbes, A. Structured light from lasers. *Laser Photonics Rev.* **13**, 1900140 (2019).

[19] Sroor, H. et al. High-purity orbital angular momentum states from a visible metasurface laser. *Nat. Photonics* **14**, 498–503 (2020).

[20] Carbone, L. et al. Generation of high-purity higher-order Laguerre-Gauss beams at high laser power. *Phys. Rev. Lett.* **110**, 251101 (2013). 23829725 10.1103/PhysRevLett.110.251101

[21] Kozawa, A., Ito, Y. & Sato, S. Generation of hollow scalar and vector beams using a spot-defect mirror. *J. Opt. Soc. Am. A Opt. Image Sci. Vis.* **27**, 2072–2077 (2010). 20808418 10.1364/JOSAA.27.002072

[22] Qiao, Z. et al. Generating high-charge optical vortices directly from laser up to 288th order. *Laser Photonics Rev.* **12**, 1800019 (2018).

[23] Sheng, Q. et al. Ultra-high-order Laguerre–Gaussian field generated directly from a laser cavity with spherical aberration. *Laser Photonics Rev.* **17**, 2300369 (2023).

[24] Kogelnik, H. & Li, T. Laser beams and resonators. *Appl. Opt.* **5**, 1550–1567 (1966). 20057590 10.1364/AO.5.001550

[25] Fox, A. G. & Li, T. Resonant modes in a maser interferometer. *Bell Syst. Tech. J.* **40**, 453 (1961).

[26] Laabs, H. & Ozygus, B. Excitation of Hermite Gaussian modes in end-pumped solid-state lasers via off-axis pumping. *Opt. Laser Technol.* **28**, 213–214 (1996).

[27] Chen, Y. F. et al. Generation of Hermite-Gaussian modes in fiber-coupled laser-diode end-pumped lasers. *IEEE J. Quantum Electron.* **33**, 1025–1031 (1997).

[28] Shen, Y. et al. Wavelength-tunable Hermite–Gaussian modes and an orbital-angular-momentum-tunable vortex beam in a dual-off-axis pumped Yb: CALGO laser. *Opt. Lett.* **43**, 291–294 (2018). 29328262 10.1364/OL.43.000291

[29] Gutiérrez-Cuevas, R. & Alonso, M. A. Modal Majorana sphere and hidden symmetries of structured-Gaussian beams. *Phys. Rev. Lett.* **125**, 123903 (2019). 10.1103/PhysRevLett.125.123903 33016748

[30] Gutiérrez-Cuevas, R. et al. Generalized Gaussian beams in terms of Jones vectors. *J. Opt.* **21**, 084001 (2019).

[31] Li, C. Y. et al. Modal interface for structured light via liquid-crystal planar optics. *Phys. Rev. Appl.* **21**, 034021 (2024).

[32] Wu, H. J. et al. Observation of anomalous orbital angular momentum transfer in parametric nonlinearity. *Phys. Rev. Lett.* **130**, 153803 (2023). 37115865 10.1103/PhysRevLett.130.153803

[33] Cao, Z. et al. Talbot-like pattern evolution in complex structured light from a unitary transformation. *Opt. Express* **32**, 28025–28034 (2024). 39538626 10.1364/OE.530909

[34] Yu, B. et al. Single-shot full characterization of the spatial wavefunction of light fields via stokes tomography. *Appl. Sci.* **14**, 2067 (2024).

[35] Giordmaine, J. A. & Miller, R. C. Tunable coherent parametric oscillation in LiNbO_3_ at optical frequencies. *Phys. Rev. Lett.* **14**, 973 (1965).

[36] Pask, H. M. The design and operation of solid-state Raman lasers. *Prog. Quantum Electron.* **27**, 3–56 (2003).

[37] Merklein, M. et al. 100 years of Brillouin scattering: historical and future perspectives. *Appl. Phys. Rev.* **9**, 041306 (2022).

[38] Mei, H. et al. Amplification of light pulses with orbital angular momentum in nitrogen ions lasing. *Opt. Express* **31**, 31912–31921 (2023). 37859005 10.1364/OE.500041

[39] Shen, B. et al. High-order vortex air lasing generated by plasma amplification. *Laser Photonics Rev.* **19**, 2401276 (2025).

[40] Liu, H. et al. Generation of femtosecond polygonal optical vortices from a mode-locked quasi-frequency-degenerate laser. *Light Sci. Appl.* **14**, 222 (2025). 40545506 10.1038/s41377-025-01902-1 PMC12183311

[41] Shen, Y. et al. Polygonal vortex beams. *IEEE Photonics J.* **10**, 1–16 (2018).

[42] Sugic, D. et al. Particle-like topologies in light. *Nat. Commun.* **12**, 6785 (2021). 34811373 10.1038/s41467-021-26171-5 PMC8608860

[43] Zhong, R. Y. et al. Gouy-phase-mediated propagation variations and revivals of transverse structure in vectorially structured light. *Phys. Rev. A* **103**, 053520 (2021).

[44] Gao, S. et al. Paraxial skyrmionic beams. *Phys. Rev. A* **104**, 049901 (2021).

[45] Shen, Y. et al. Free-space topological optical textures: tutorial. *Adv. Opt. Photonics* **17**, 295–374 (2025).

[46] Wang, J. et al. Vectorial light–matter interaction: exploring spatially structured complex light fields. *AVS Quantum Sci.* **2**, 031702 (2020).

