# Supplementary material for: Tunable structured laser over full spatial spectrum
Source: Light Sci Appl. 2026 Mar 13;15:169. doi: 10.1038/s41377-026-02243-3 (PMC12982750; doi:10.1038/s41377-026-02243-3)
Supplement: Supplementary file 1 — Supplementary Document [file 41377_2026_2243_MOESM1_ESM.pdf]

Supplementary Information for

Tunable Structured Laser over Full Spatial Spectrum

Quan Sheng[1,2], Jing-Ni Geng[1], Jia-Qi Jiang[2,3], Tian-Chang Liu[1], David James Spence[4], Helen Margaret Pask[4], Allam Srinivasa Rao[5], Takashige Omatsu[1,5], Shi-Jie Fu[1], Zhen-Xu Bai[6], Zhi-Wei Lv[6], Wei Shi[1,*], Jian-Quan Yao[1,3], Carmelo Rosales-Guzmán[2,3,7], and Zhi-Han Zhu[2,3*]

[1] State Key Laboratory of Precision Measuring Technology and Instruments, School of Precision Instrument of Opto-Electronics Engineering, Tianjin University, Tianjin, 300072, China
[2] Institute of Quantum Science and Technology, and Department of Physics, Yanbian University, Yanji, Jilin 133002, China
[3] Wang Da-Heng Center, Heilongjiang Key Laboratory of Quantum Control, Harbin University of Science and Technology, Harbin,150080, China
[4] MQ Photonics Research Centre, School of Mathematical and Physical Sciences, Macquarie University, Sydney, NSW 2109, Australia
[5] Molecular Chirality Research Center, Chiba University, 1-33 Yayoi-cho, Inage-ku, Chiba 263-8522, Japan
[6] Center for Advanced Laser Technology, Hebei University of Technology, Tianjin 300401, China
[7] Centro de Investigaciones en Óptica, A.C., Loma del Bosque 115, Colonia Lomas del Campestre, 37150 León, Gto., Mexico
[*] e-mail: shiwei@tju.edu.cn, and zhuzhihan@ybu.edu.cn

Theoretical details

1. Hermite-Laguerre-Gauss modes

The transverse structure of a paraxial light field and its corresponding evolution upon propagation can fully be described by a solution of the paraxial wave equation (PWE), which can be regarded as an analog of the Schrödinger equation for free particles. Except for an ideal plane wave, any eigensolution of the wave equation corresponds to a physically realizable "eigen" spatial mode, whose transverse structures are propagation invariant apart from an overall change of size. For instance, the well-known Laguerre-Gauss (LG) and the Hermite-Gauss (HG) modes are eigensolutions of the PWE in cylindrical and Cartesian coordinates, respectively, and their complex amplitude are [1,2]

$$\mathrm{LG}_{\ell,p}(r,\varphi,z)=\sqrt{\frac{2p!}{\pi(p+|\ell|)!}}\frac{1}{w(z)}\left(\frac{\sqrt{2}r}{w(z)}\right)^{|\ell|}\cdot \mathrm{e}^{-\frac{r^2}{w_z^2}}\cdot L_p^{|\ell|}\left(\frac{2r^2}{w_z^2}\right)e^{i\left[kz+\frac{kr^2}{2R_z}+\ell\varphi-(N+1)\phi\right]} \tag{S1}$$

$$\mathrm{HG}_{m,n}(x,y,z)=\sqrt{\frac{2^{1-n-m}}{\pi n!\,m!}}\frac{1}{w(z)}\cdot \mathrm{e}^{-\frac{r^2}{w_z^2}}\cdot H_n\left(\frac{\sqrt{2}x}{w_z}\right)H_m\left(\frac{\sqrt{2}y}{w_z}\right)e^{i\left[kz+\frac{kr^2}{2R_z}-(N+1)\phi\right]} \tag{S2}$$

where $z_R=kw_0^2/2$, $w_z=w_0\sqrt{1+(z/z_R)^2}$, and $R_z=z^2+z_R^2/z$ are the Rayleigh length, beam waist, and radius of curvature, respectively. $L_p^{|\ell|}(\cdot)$ and $H_{m/n}(\cdot)$ denote the Laguerre and Hermite polynomials, respectively. $\phi=\arctan(z/z_R)$ is the Gouy phase upon propagation; and their modal orders are defined as $N=2p+|\ell|=m+n$.

Above Hermite- and Laguerre-Gaussian mode families have their own special features in transverse structures but have the same fundamental TEM_{00} mode, and each can independently constitute a unique and unbounded 2D Hilbert space. Therefore, in principle, we can represent any Gaussian mode by using coherent superpositions (i.e., complex series) of LG, HG, or their intermediate modes. In this work, for the convenience of calculating orbital angular momentum (OAM), LG modes and associated azimuthal index (ℓ) spectra were used to analyze the OAM of beams.

2. SU(2) modal extension on the HLG modal sphere

As a type of spatially structured Gaussian modal family exhibiting SU(2) symmetry, generalized Hermite-Laguerre-Gauss (HLG) modes establish a close connection between HG modes and LG modes via the geometric representation with unitary modal sphere, and its spatial wavefunction (or complex amplitude) can be expressed as the superposition of HG modes [3,4]

$$\mathrm{HLG}_{m,n}^{(\phi,\theta)}(x,y,z)=\sum_{s=0}^{N} D_{m,s}^{(N/2)}(\phi,\theta)\cdot \mathrm{HG}_{s,N-s}(x,y,z) \tag{S3}$$

where (ϕ,θ) represents the longitude and latitude angles of the state on the unitary modal sphere, the superposition coefficients are given by the Wigner D-function:

$$D_{m,s}^{\left(\frac{N}{2}\right)}(\phi,\theta)=e^{-im\beta(\phi,\theta)}\sqrt{s!\,(N-s)!\,m!\,(N-m)!}\,*\sum_{v=\max(0,s-m)}^{\min(s,N-m)}(-1)^{v}\frac{\left[\cos\left(\frac{\alpha(\phi,\theta)}{2}\right)\right]^{N-m+s-2v}\left[\sin\left(\frac{\alpha(\phi,\theta)}{2}\right)\right]^{m-s+2v}}{(N-m-v)!\,v!\,(s-v)!\,(m-s+v)!} \tag{S4}$$

Here the SU(2) rotation factors $\alpha(\phi,\theta)$ and $\beta(\phi,\theta)$ are given by

$$\alpha(\phi,\theta)=\cos^{-1}\left[\cos\phi\cdot\sqrt{(\cos\theta)^2}\right] \tag{S5}$$

$$\beta(\phi,\theta)=-\sin^{-1}\left[\frac{\sin\theta}{\sqrt{1-(\cos\phi)^2(\cos\theta)^2}}\right] \tag{S6}$$

Fig. S1 examples three such spheres, in particular, the last two are theoretical references for experimental data shown in Fig. 4(c).

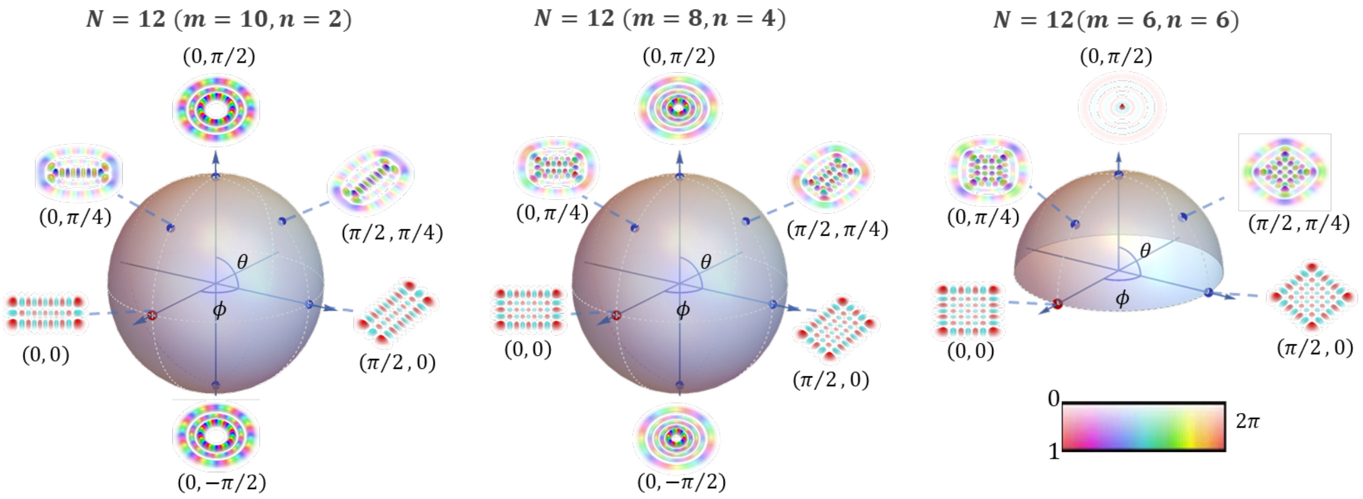

Figure S1. Theoretical examples of HLG modes on sphere simulated using Eq. (S3).

3. HG modal gain and selection via off-axis pumping

The essence of mode selection is to introduce mode-varying gain or loss, ensuring the target mode has a net gain just reaches threshold while those of other modes do not. The threshold pump power of an end-pumped solid-state laser is given by the follow equations. The essence of mode selection is to introduce mode-varying gain or loss, ensuring the target mode has a net gain just reaches

threshold while those of other modes do not. The threshold pump power of an end-pumped solid-state laser is given by the follow equations [5]

$$P_{\mathrm{th}} = \frac{\gamma I_{\mathrm{sat}}}{\eta_p L}\frac{1}{J} \tag{S7}$$

$$J = \int_V r(x,y,z)s(x,y,z)dV \tag{S8}$$

where γ is the total logarithmic loss per pass, I_{sat} is the saturation intensity (~830 W cm^{-2} for a-cut Nd:YVO$_4$ crystal at the 1064.3 nm emission peak), L is the length of the active medium (8 mm), η_p is pump efficiency (65%) with fractional absorption (86% for the crystal used under 808 nm pump) and quantum defect (24% for 808 nm pump and 1064 nm laser) considered. J is the overlap integral between the pump beam and the cavity mode, where $r(x,y,z)$ and $s(x,y,z)$ are normalized intensity distribution of the cavity mode and pump beam, respectively, in the gain medium. Since different transverse modes have different intensity profiles, their overlap integrals J with the pump beam are different, and the mode overlaps best with the pump beam would have the lowest threshold and win the competition among a family of eigenmodes of the cavity. Taking $\mathrm{HG}_{4,0}^{0°}$ with its intensity peaks $\Delta x = 1.75w_0$ as an example, when the pump beam with a $wp = 0.7w_0$ is aligned to the intensity peak of $\mathrm{HG}_{4,0}^{0°}$, the pump overlap of $\mathrm{HG}_{4,0}^{0°}$ is 1.19 and 1.31 times, respectively, of those of its neighboring modes $\mathrm{HG}_{3,0}^{0°}$ and $\mathrm{HG}_{5,0}^{0°}$. Therefore, the oscillating mode can be controlled by changing the pump position.

4. Establishing process of intracavity spatial wavefunctions

To theoretically analyze the transverse mode selection and tunability for the cavity design, i.e., the establishing process of intracavity spatial wavefunctions, we employed the cascading calculation of noise-initialed diffraction in the cavity with off-axis pump gain. Such numerical method is also known as Fox-Li iteration [6-9]. Specifically, the formula of the intracavity diffraction integral in the spatial domain can be expressed as

$$E_2(x,y) = \sqrt{T}\exp[g_0 P(x,y)]\left(-\frac{\mathrm{i}}{\lambda\sqrt{|B|}}\right)\exp(\mathrm{i}kL_0)\iint E_1(x',y')$$

$$\exp\left\{\frac{\mathrm{i}k}{2}\begin{bmatrix}x'\\y'\\x\\y\end{bmatrix}^{\mathrm{T}}\begin{bmatrix}B^{-1}A & -B^{-1}\\ C-DB^{-1}A & DB^{-1}\end{bmatrix}\begin{bmatrix}x'\\y'\\x\\y\end{bmatrix}\right\}\mathrm{d}x'\mathrm{d}y \tag{S9}$$

where E_1 and E_2 are the light field distributions at M1 before and after a round trip, T is the transmittance of the output mirror, g_0 is the gain coefficient, and k is the wave number, $P(x,y)$ is the pump field distribution in the gain medium. According to the astigmatic configuration in this study, the $[A,B;C,D]$ matrix used here is extended to a 4×4 form, obtained from the resonant cavity transmission matrix, given by

$$\begin{bmatrix} A & B \\ C & D \end{bmatrix} = \begin{bmatrix} 1 & 0 & L_1 & 0 \\ 0 & 1 & 0 & L_1 \\ 0 & 0 & 1 & 0 \\ 0 & 0 & 0 & 1 \end{bmatrix} \begin{bmatrix} 1 & 0 & 0 & 0 \\ 0 & 1 & 0 & 0 \\ -\frac{2}{R\cos\theta} & 0 & 1 & 0 \\ 0 & -\frac{2\cos\theta}{R} & 0 & 1 \end{bmatrix} \begin{bmatrix} 1 & 0 & L_2 & 0 \\ 0 & 1 & 0 & L_2 \\ 0 & 0 & 1 & 0 \\ 0 & 0 & 0 & 1 \end{bmatrix}$$

$$\begin{bmatrix} 1 & 0 & L_2 & 0 \\ 0 & 1 & 0 & L_2 \\ 0 & 0 & 1 & 0 \\ 0 & 0 & 0 & 1 \end{bmatrix} \begin{bmatrix} 1 & 0 & 0 & 0 \\ 0 & 1 & 0 & 0 \\ -\frac{2}{R\cos\theta} & 0 & 1 & 0 \\ 0 & -\frac{2\cos\theta}{R} & 0 & 1 \end{bmatrix} \begin{bmatrix} 1 & 0 & L_1 & 0 \\ 0 & 1 & 0 & L_1 \\ 0 & 0 & 1 & 0 \\ 0 & 0 & 0 & 1 \end{bmatrix} \quad \text{(S10)}$$

where L_1 and L_2 are the lengths of M1-M2 and M2-M3 respectively, R is the radius of curvature of M2, and θ is the folding half angle of M2. Consider the pump beam was guided by and outputted from a multimode fiber, the pump field distribution is thus assumed to be a flat-top profile, expressed as

$$P(x,y) = \Theta\left[w_p^2 - (x - \Delta x)^2 - (y - \Delta y)^2\right] \quad \text{(S11)}$$

where Θ is a step function, w_p is the pump light radius, and w_p is set to 0.7 times the fundamental mode spot radius w in the calculation with reference to the actual experimental parameters; Δx and Δy are the displacements of the pump light relative to the resonant cavity optical axis in the x-axis (horizontal) and y-axis (vertical) directions, respectively. To simplify the calculation, the divergence of the pump light, other passive losses except the coupled output, and the refractive index of the laser crystal and the influence of the thermal effect therein were ignored in the above process. For instance, Figure S2 shows the simulated establishment process of intracavity spatial wavefunctions under the off-axis pump gain $\Delta x = \Delta y = 1.75w$, where up- and down rows exhibit results output from cavities with and without astigmatic detuning, respectively.

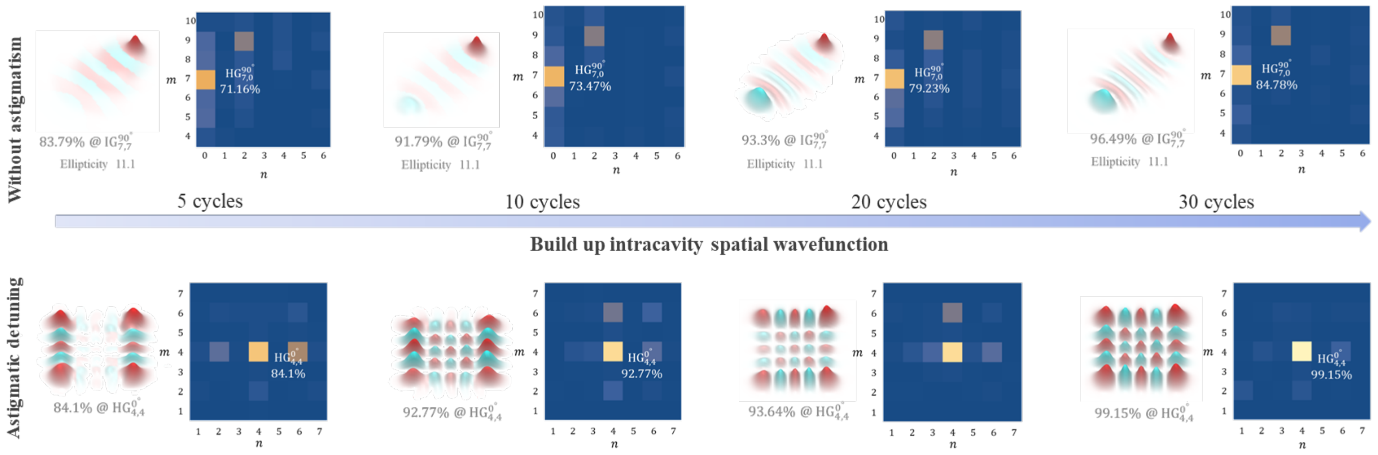

Figure S2. Simulated evolution of intracavity spatial wavefunctions via the Fox-Li iteration.

Experimental details

1. Cavity design

The V-shaped cavity folded by a concave folding mirror CM which introduced astigmatism was used in the experiment for 2D HG mode laser generation. The flat IM and concave CM were coated for highly-reflective (R>99.9%) at the 1064 nm laser wavelength and highly-transmissive (T>98%) at the 878.6 nm pump wavelength. With the distances IM-CM and CM-OC being 130 mm and 170 mm and a half fold angle $\delta = 10°$, the intracavity fundamental mode beam size was calculated using $ABCD$ matrix, as plotted in Fig. S3. The beam radii at the mirrors are 136 μm×137 μm, 342 μm×340 μm, and 219 μm×229 μm, respectively. With the fundamental mode beam size determined, the intensity profiles of the high-order $HG_{m,n}$ mode beams can be calculated using the Eq. (1). Then their overlap integral with the pump beam, as well as the maximum mode order allowed by the limited apertures of the elements, can be calculated. Figure S4 shows the contour plot of the fractional energy blocked (diffraction loss) of the $HG_{m,m}$ beam by a quadrate aperture. The 5 mm ×5 mm crystal aperture, which is ~36.5 times of fundamental mode beam radius in the crystal, allows $HG_{300,300}$ mode with single-pass loss as low as 0.005%. Meanwhile, the three cavity mirrors with the same aperture of 25.4 mm would not block the beam.

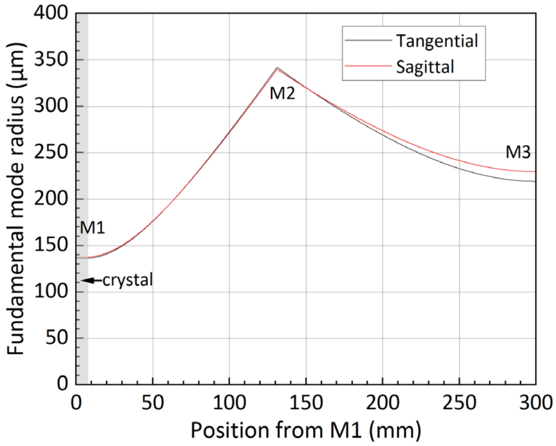

Figure S3. Calculated fundamental mode beam radius in the laser cavity.

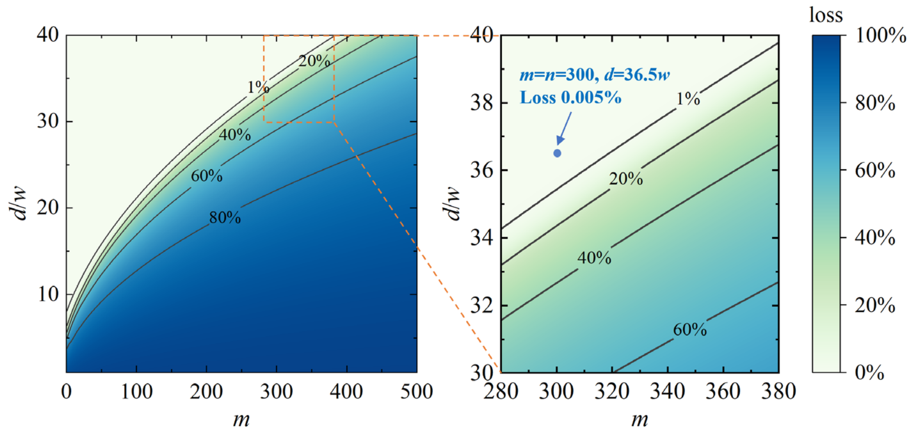

Figure S4. Calculated energy blocked by a quadrate aperture on different orders of $HG_{m,n}$ mode beams with $m = n$.

2. Spatial complex amplitude Characterization

In order to measure the full spatial structure of three interacting beams, we built a complex amplitude profiler based on spatial Stokes tomography, and Fig. S5 shows the schematic of the device. The measured beam with horizontal polarization, denoted as $E(\vec{r})\hat{e}_H$, was combined with a vertical polarized ancillary Gaussian beam, denoted as $\mathrm{HG}_{0,0}(\vec{r})\hat{e}_V$, at a polarizing beam splitter (PBS) and, in consequence, generating a vector beam, i.e., $\boldsymbol{E}(\vec{r}) = 1/\sqrt{2}\left[E(\vec{r})\hat{e}_H + \mathrm{HG}_{0,0}(\vec{r})\hat{e}_V\right]$. Then, a non-polarizing beam splitter (NPBS) separates the vector beam into four equal-amplitude ones, which are further filtered by four polarizers set to passing H, V, D and L polarizations, respectively. Diagram in Fig. S5 righthand shows details of the NPBS made by liquid-crystal geometric phase and an integrated polarizing filter for four output ports, see Ref. [9] for more details. Theses polarization projections can be regarded as spatial-resolved Stokes measurement, i.e., $\langle \boldsymbol{E}(r,\varphi)|\hat{\sigma}_{0\text{-}3}^{\mathrm{spin}}(\vec{r})|\boldsymbol{E}(r,\varphi)\rangle$, where $\hat{\sigma}_{0\text{-}3}^{\mathrm{spin}}(\vec{r})$ is spatial-resolved Pauli matrices with respect to the spin space. In this way, the corresponding mutually unbiased observables become spatial Stokes distributions $S_{0\text{-}3}(r,\varphi)$, with which we can reconstruct the polarization distribution of the vector mode. Beyond the polarization pattern, more importantly, we can also obtain the spatial polarization orientation, or spatial intramodal phase, of the vector mode, given by

$$\gamma(\vec{r}) = \frac{1}{2}\tan^{-1}\left(\frac{S_2(\vec{r})}{S_1(\vec{r})}\right) \tag{S12}$$

Because the ancillary beam was a Gaussian beam, separated from signal beam via pinhole filtering, near waist plane that has a flat wavefront, therefore, Eq. (S5) is the wavefront of the beam to be measured. Combined with directly observable beam profile, we obtained the complex amplitude of measured beam $E(\vec{r})$. This spatial wavefunction has recorded full knowledge about the laser beam, thus, we can calculate its modal purity and beam quality M^2 by using numerical projection and diffraction integral, respectively, see Ref. 9 for more details about this technique.

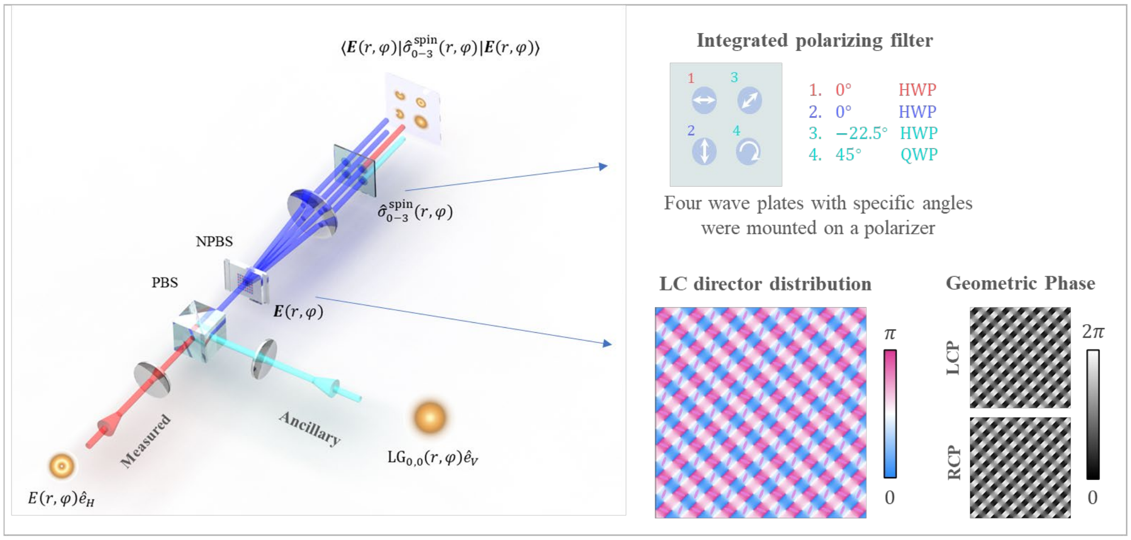

Figure S5. Complex amplitude profiler based on spatial Stokes tomography. Righthand diagram shows details of the nonpolarizing beam splitter based on liquid-crystal geometric phase and the integrated polarizing filter.

Additional Data

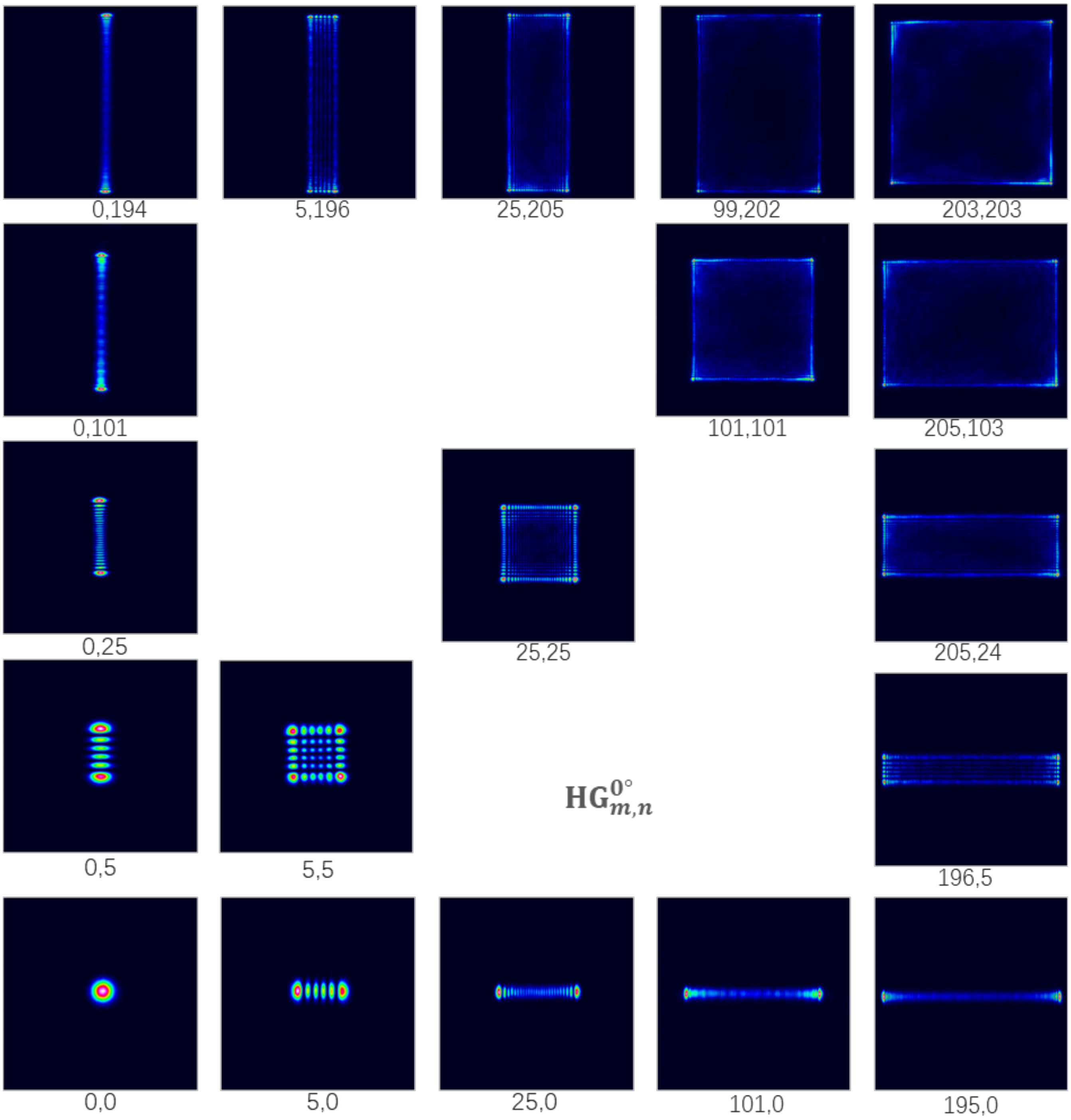

Figure S6. Intensity profiles of spatial complex amplitudes shown in Fig. 4(a) in the main text.

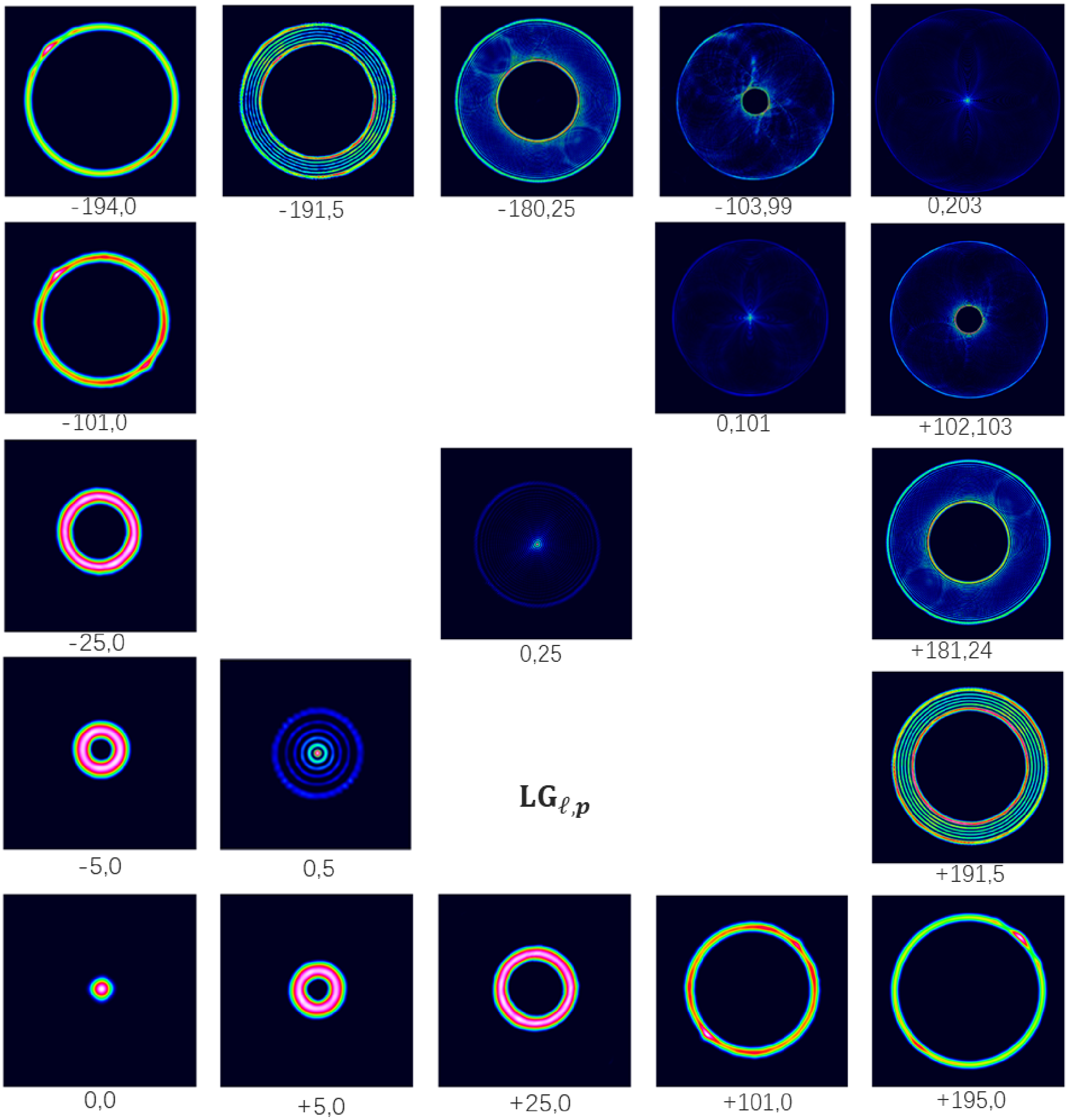

Figure S7. Intensity profiles of spatial complex amplitudes shown in Fig. 4(b) in the main text.

References

1. Allen, L. et al. Orbital angular momentum of light and the transformation of Laguerre-Gaussian laser modes. *Physical Review A* **45**, 8185-8189 (1992).

2. Beijersbergen, M. W. et al. Astigmatic laser mode converters and transfer of orbital angular momentum. *Optics Communications* **96**, 123-132 (1993).

3. Dennis, M. R. & Alonso, M. A. Swings and roundabouts: optical Poincaré spheres for polarization and Gaussian beams. *Philosophical Transactions of the Royal Society A: Mathematical, Physical and Engineering Sciences* **375**, 20150441 (2017).

4. Gutiérrez-Cuevas et al. Generalized Gaussian beams in terms of Jones vectors. *Journal of Optics* **21**, 084001 (2019).

5. Chen, Y. F. et al. Generation of Hermite-Gaussian modes in fiber-coupled laser-diode end-pumped lasers. *IEEE Journal of Quantum Electronics* **33**, 1025-1031 (1997).

6. Fox, A. G. & Li, T. Y. Resonant modes in a maser interferometer. *The Bell System Technical Journal* **40**, 453-488 (1961).

7. Arnaud, J. A. Nonorthogonal optical waveguides and resonators. *The Bell System Technical Journal* **49**, 2311-2348 (1970).

8. Longhi, S. Fractional Schrödinger equation in optics. *Optics Letters* **40**, 1117-1120 (2015).

9. Yu, B. S. et al. Single-shot full characterization of the spatial wavefunction of light fields via stokes tomography. *Applied Sciences* **14**, 2067 (2024).
